# Surgical Repair of a Giant Aberrant Artery Aneurysm in an Adult with Type B Interrupted Aortic Arch

**DOI:** 10.5761/atcs.cr.25-00205

**Published:** 2026-02-13

**Authors:** Kaoutar Farahi, Ramzi Abi Akar, Francesca Pitocco, Paul Achouh, Bastien Poitier

**Affiliations:** 1 Cardiovascular Surgery Department, AP-HP, Georges Pompidou European Hospital, Paris, France; 2 Department of Radiology, Cardiovascular Imaging Unit, AP-HP, Georges Pompidou European Hospital, Paris, France

**Keywords:** interrupted aortic arch, aneurysm, surgical repair

## Abstract

Interrupted aortic arch (IAA) is a rare congenital anomaly usually diagnosed in infancy and associated with intracardiac defects, making survival into adulthood without repair exceptional. We report the case of a 62-year-old woman with longstanding hypertension who presented with progressive dyspnea and dysphagia. Computed tomography angiography identified a type B IAA associated with a giant 96-mm aneurysm arising from an aberrant retroesophageal artery connecting the right subclavian artery to the descending thoracic aorta, without associated cardiac abnormalities. Surgical management consisted of isolated resection of the aneurysmal segment and placement of a 14-mm Dacron graft, without reconstruction of the aortic arch given the patient’s age and stable hypertension. The postoperative course was uneventful, and follow-up imaging showed stable repair with complete symptom resolution. This case highlights the possibility of long-term survival without restoration of normal aortic anatomy, and suggests that a tailored, complication-focused surgical approach may be appropriate in selected adult patients.

## Abbreviations

IAA
: interrupted aortic arch
CTA
: computed tomography angiography
CPB
: cardiopulmonary bypass

## Introduction

Interrupted aortic arch (IAA) is a rare congenital anomaly defined by a complete anatomical discontinuity between the ascending and descending thoracic aorta. It represents less than 1% of all congenital heart defects, with over 97% of cases involving additional cardiac abnormalities: ventricular septal defect in 80%–90%, common arterial trunk in 10%, and aortopulmonary window in 5%–10%. Bicuspid aortic valve (60%), subaortic stenosis (20%), and DiGeorge syndrome (33%–50%) are also commonly reported.^1)^ Typically diagnosed in infancy and associated with an extremely poor prognosis without surgical intervention, its occurrence in adulthood is exceptionally rare.^1–3)^

The most widely used classification, proposed by Celoria and Patton, categorizes IAA into three types based on the site of interruption: type A (distal to the left subclavian artery), type B (between the left common carotid and left subclavian arteries), and type C (between the innominate and left common carotid arteries). Type B is the most common in neonates, whereas type A predominates in adult cases.^4,5)^

Adult cases may be asymptomatic or present with systemic hypertension, limb blood pressure discrepancies, claudication, headaches, limb swelling, heart failure, or complications like aortic dissection.^3,5,6)^ Diagnosis relies on both computed tomography angiography (CTA) and echocardiography; while echocardiography provides useful information at the initial assessment, CTA is widely regarded as the gold standard imaging modality.^7,8)^

## Case Report

We present the case of a 62-year-old woman with a longstanding history of hypertension, referred to our center for management of a type B IAA associated with a giant aneurysm of an aberrant retroesophageal artery connecting the right subclavian artery and descending aorta, but no intracardiac anomalies. Her primary symptoms included progressive dyspnea over the past 3 years, intermittent upper back pain, and dysphagia that began 1 year prior to presentation and had gradually worsened. Of note, she had previously experienced two successful and uneventful pregnancies.

**Fig. 1:**
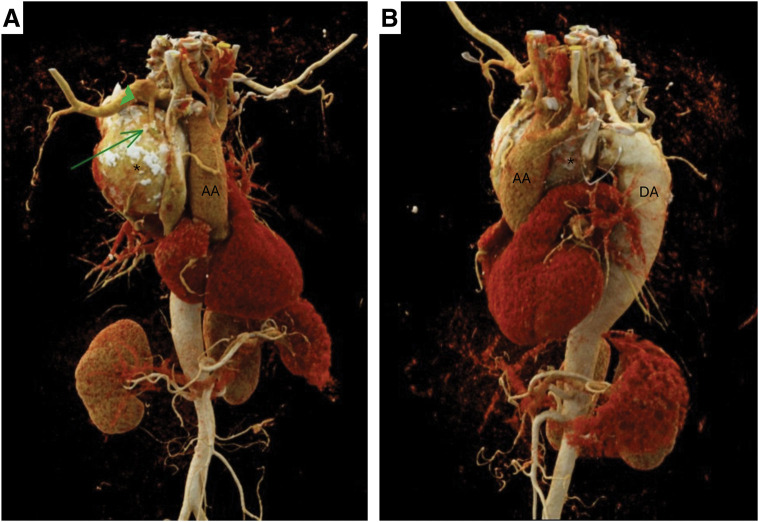
(**A** and **B**) Volume Rendering Reconstruction showing the anatomy of the aorta: green arrow showing the vascular connection between the aneurismal artery (*) and the right subclavian artery (green arrow head). AA: ascending aorta; DA: descending aorta

**Fig. 2:**
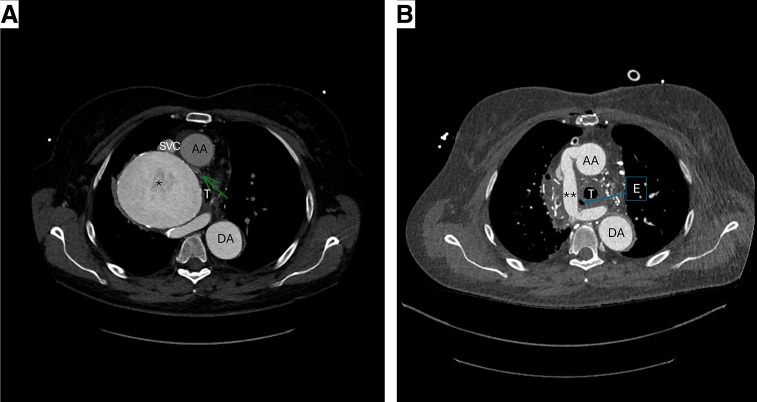
(**A**) Preoperative CTA showing the giant aneurysm of the aberrant retroesophageal artery (*) connecting the ascending and descending aorta responsible for the brachiocephalic left trunk compression (green arrow). (**B**) “Postoperative CTA demonstrating the Dacron graft (**) anastomosed between the ascending and descending aorta. AA: ascending aorta; DA: descending aorta; SVC: superior vena cava; T: trachea; E: esophagus; CTA: computed tomography angiography

On physical examination, she was hypertensive despite treatment with a combination of beta-blockers, angiotensin II receptor blockers, and diuretics. Cardiac and pulmonary auscultation were unremarkable. Transthoracic echocardiography revealed a non-dilated, non-hypertrophied left ventricle with preserved global and segmental systolic function, and no valvular abnormalities. CTA was performed with a 128-slice dual-source computed tomography (Somatom Force Siemens Healthineers, Forchheim, Germany), and confirmed a type B IAA associated with a large aneurysm measuring 96.1 mm, arising from an aberrant retroesophageal artery originating from the right subclavian artery and connecting to the descending thoracic aorta (**Figs. 1** and **2A**). Before crossing the midline, the aberrant artery returned to a normal diameter (13 mm). No additional collateral vessel hypertrophy was identified. Additionally, the innominate vein was abnormally positioned, coursing underneath the ascending aorta and displacing the superior vena cava anteriorly. Diameters of the aortic root structures were within normal range.

Following multidisciplinary discussion, the patient underwent isolated surgical replacement of the aneurysmal segment of the aberrant artery, without aortic arch reconstruction. The surgical strategy was carefully selected considering several unique clinical features, including the very late onset of symptoms, the absence of additional congenital cardiac anomalies, and her longstanding but well-controlled hypertension. Notably, her history of two successful and uneventful pregnancies is indicative of significant physiological adaptation. Surgery was performed under general anesthesia through a standard median sternotomy. Intraoperative transesophageal echocardiography confirmed the absence of septal abnormalities. Cardiopulmonary bypass (CPB) was established using double arterial cannulation (ascending aorta and right femoral artery) to ensure adequate perfusion during clamping of the aberrant artery, and double venous cannulation. The aneurysmal retroesophageal artery was partially obscured by the superior vena cava (SVC), which lay anterior to the aneurysm. Because the innominate vein coursed posterior to the aortic arch, mobilization of the SVC to improve surgical exposure was limited. Consequently, transection of the SVC was required to allow safe access to the aneurysmal artery. The aberrant retroesophageal artery was then carefully dissected and controlled proximally and distally. The right subclavian artery was clamped proximal and distal to the origin of the aberrant vessel, which was subsequently resected. The aneurysmal segment was replaced with a 14-mm Dacron graft, with the proximal anastomosis performed directly to the ascending aorta under lateral clamping. Continuity of the right subclavian artery was restored via end-to-end anastomosis after resection of the aberrant origin. Finally, the SVC was reconstructed and CPB was discontinued uneventfully after 185 minutes.

Postoperatively, the patient exhibited no upper-lower limb pressure gradient or neurological deficits. Her initial recovery was uneventful. She was transferred to the ward on postoperative day 3 and discharged home on postoperative day 10. She was readmitted on postoperative day 23 for percutaneous drainage of a left pleural effusion. Follow-up CTA demonstrated a patent retroesophageal ascending-to-descending aortic bypass graft with no evidence of perianastomotic complications (**Fig. 2B**). At the 3-month follow-up visit, the patient reported complete resolution of dysphagia and improved exercise tolerance, as she was now able to perform daily activities without experiencing dyspnea. At 19 months post-surgery, she continued to engage in regular physical activity with excellent tolerance and no functional limitation. At this follow-up, control CTA demonstrated a stable surgical repair, with a patent graft and no signs of disease progression.

## Discussion

Surgical correction remains the gold-standard treatment for IAA and may be performed via a single-stage complete repair or a staged approach depending on associated defects.^9–11)^ However, in some patients, the progressive development of extensive collateral circulation can ensure sufficient distal perfusion for many years, thereby delaying the diagnosis into adulthood. Imaging and hemodynamic modeling have improved insight into collateral physiology in chronic aortic obstruction, demonstrating that enlarged systemic collaterals can sustain distal perfusion^6,12)^ but may also induce areas of elevated wall shear stress and abnormal pressure loading, thereby predisposing selected vessels to aneurysm formation or increasing risk of aortic dissection.^13)^ In such cases, a more limited therapeutic strategy, ranging from optimized medical management^3)^ with antihypertensive therapy in the absence of major complications to targeted intervention for life-threatening conditions such as isolated aneurysm exclusion, may represent a safe and effective alternative to extensive aortic arch reconstruction.

In the present case, the primary clinical issue was not the interrupted aortic arch itself, but the massive aneurysmal degeneration of an aberrant retroesophageal artery, which had become the dominant collateral pathway supplying the descending aorta. Aneurysmal transformation of collateral vessels in adult IAA is uncommon. Sasaki et al.^14)^ described a 50-mm thoracic collateral aneurysm arising from intercostal circulation in a 49-year-old man with type A IAA; Patel et al.^15)^ reported a 20-mm post-interruption descending aortic aneurysm associated with a bicuspid aortic valve in a 53-year-old woman; and Shakerian et al.^16)^ documented a 76-mm right subclavian artery aneurysm associated in type B IAA. Notably, only one of these patients underwent surgical intervention via extra-anatomic bypass, whereas the others were managed conservatively with medications to reduce hypertension. None of those reports described the specific anatomical configuration observed in our patient, in whom a giant aneurysm arose from an aberrant retroesophageal artery functioning as the principal conduit to the distal aorta, creating a distinctive combination of high-flow dependence and rupture risk.

In adults, the chronicity of the interruption typically results in a long atretic segment, decreased aortic wall compliance, and dense collateral networks, rendering direct end-to-end repair impracticable and greatly increasing the complexity of any reconstructive approach.^3)^ Moreover, full arch reconstruction whether via interposition graft or extra-anatomic bypass carries additional risks, including the need for circulatory arrest, potential injury to the recurrent or phrenic nerves, and technically demanding mobilization of the distal arch or descending aorta through a left thoracotomy. The presence of exuberant collateral vessels further amplifies the risk of considerable intraoperative bleeding.^17)^ Accordingly, a tailored strategy was adopted in our case, focusing exclusively on aneurysm exclusion to prevent rupture while avoiding arch reconstruction.

## Conclusion

This case supports the notion that in selected adult IAA patients without significant perfusion deficits, restoration of normal aortic anatomy is not always necessary, and tailored surgical approaches, such as isolated aneurysm repair, can offer safe and effective outcomes. Nevertheless, careful long-term surveillance is required; in our practice, we suggest CTA follow-up every 2 years in the absence of disease progression.

## Declarations

### Data Availability

No new data were created or analyzed in this study. Data sharing is not applicable to this article.
